# Ceramides as Emerging Players in Cardiovascular Disease: Focus on Their Pathogenetic Effects and Regulation by Diet

**DOI:** 10.1016/j.advnut.2024.100252

**Published:** 2024-06-12

**Authors:** Riccardo Spaggiari, Sharon Angelini, Alessandra Di Vincenzo, Gerarda Scaglione, Sara Morrone, Veronica Finello, Sofia Fagioli, Fabiola Castaldo, Juana M Sanz, Domenico Sergi, Angelina Passaro

**Affiliations:** 1 Department of Translational Medicine, University of Ferrara, Via Luigi Borsari, Ferrara, Italy; 2 Department of Chemical, Pharmaceutical and Agricultural Sciences, University of Ferrara, Via Luigi Borsari, Ferrara, Italy

**Keywords:** ceramide, cardiovascular disease, dietary fatty acids, atherosclerosis, Western diet, Mediterranean diet

## Abstract

Impaired lipid metabolism is a pivotal driver of cardiovascular disease (CVD). In this regard, the accumulation of ceramides within the circulation as well as in metabolically active tissues and atherosclerotic plaques is a direct consequence of derailed lipid metabolism. Ceramides may be at the nexus between impaired lipid metabolism and CVD. Indeed, although on one hand ceramides have been implicated in the pathogenesis of CVD, on the other specific ceramide subspecies have also been proposed as predictors of major adverse cardiovascular events. This review will provide an updated overview of the role of ceramides in the pathogenesis of CVD, as well as their pathogenetic mechanisms of action. Furthermore, the manuscript will cover the importance of ceramides as biomarkers to predict cardiovascular events and the role of diet, both in terms of nutrients and dietary patterns, in modulating ceramide metabolism and homeostasis.

**Statement of Significance**

This review highlights the impact of nutrients as well as dietary patterns on ceramide synthesis and accumulation both in tissues and circulation. Nutrients and dietary patterns known to increase cardiovascular disease risk are also able to foster ceramide anabolism.

## Introduction

Cardiovascular diseases (CVDs) represent the leading cause of mortality worldwide [1]. The burden of CVD, particularly nowadays, is fostered by the epidemic proportions that obesity has reached globally [1]. In this context, poor diet quality and energy overconsumption play a pivotal role. Particularly, the intake of ultra-processed foods, as typically occurs as part of the Western diet, and a sedentary lifestyle have been key in promoting obesity and its comorbidities, including CVD. In this regard, the derangements in lipid metabolism and circulating lipid profile are crucial in dictating the effect of diet and obesity on CVD pathogenesis. Currently, the main clinical lipid biomarker used as a predictor of CVD is represented by cholesterol associated with cholesterol associated with low-density lipoproteins (LDL-cholesterol). However, emerging scientific evidence points toward circulating ceramides as potential novel lipid biomarkers for cardiometabolic risk [2]. Ceramides, and their derivatives, are a subclass of sphingolipids whose structure encompasses a sphingosine base and a fatty acid chain of variable length, joined together by an amide bond. Ceramides are synthesized by virtually all types of cells via 3 different pathways: de novo synthesis, where serine-palmitoyl transferase (SPT) 1 and 2 and ceramide synthase (CerS) are key enzymes, the sphingomyelinase (SMase) pathway, which implies the action of either acid or neutral SMase and the salvage pathway, all extensively described elsewhere [[3], [4], [5]]. These sphingolipids are involved in several physiological processes, being components of cellular and mitochondrial membranes and interacting with key pathways in lipid and glucose homeostasis [6]. In particular, ceramides embedded in the plasma membranes as part of lipid rafts modulate the distribution of surface receptors and stability of cell membranes [7]. In addition, ceramides are bioactive lipids and, as such, are able to modulate a wide array of intracellular signaling pathways related to cell growth, apoptosis, senescence, and differentiation [8,9]. In consideration of their role as bioactive lipids, it follows that alterations in their circulating and tissue levels may impact health. Indeed, the disruption of ceramide homeostasis and their buildup in the circulation as well as in metabolically active tissues and the vascular endothelium has been associated with impaired cardiometabolic health. In keeping with this, specific ceramide subspecies have been proposed as potential biomarkers and putative effectors of CVD and type 2 diabetes (T2D) [10,11].

This narrative review aims at providing an updated overview of the role of ceramides as novel biomarkers of CVD, which can potentially be translated to the clinic. In addition, it will highlight the involvement of ceramides in the pathogenesis of CVD and the role of diet in modulating the levels and type of these sphingolipids, both in the circulation and tissues.

## Ceramides: A New Player in Cardiovascular Disease

The pathogenesis of atherosclerosis is the result of a complex interplay between metabolic and inflammatory risk factors. Despite cholesterol, in particular, LDL-cholesterol , carrying most of the blame as a driver of CVD, ceramides are emerging as a novel independent factor able to predict cardiovascular disease risk [12]. Indeed, the crucial role of ceramides in the pathophysiology of CVD has been described both in animal and human studies [13], with peculiar ceramide subspecies (C16:0, C18:0, C24:0, and C24:1) being reported as independent predictors of cardiovascular events [14,15].

Ceramides appear to play a direct role in promoting atherosclerosis. In this regard, ceramides have been detected in atherosclerotic plaques where they facilitate the transport and entry of oxidized LDL-cholesterol into intimal cells, the initial step in atheroma formation [10]. Moreover, the tendency of oxidized LDL-cholesterol to aggregate is influenced by the lipidome of these lipoproteins themselves, with an increase in ceramide content in LDL enhancing their aggregation capacity [16].

**FIGURE 1:**
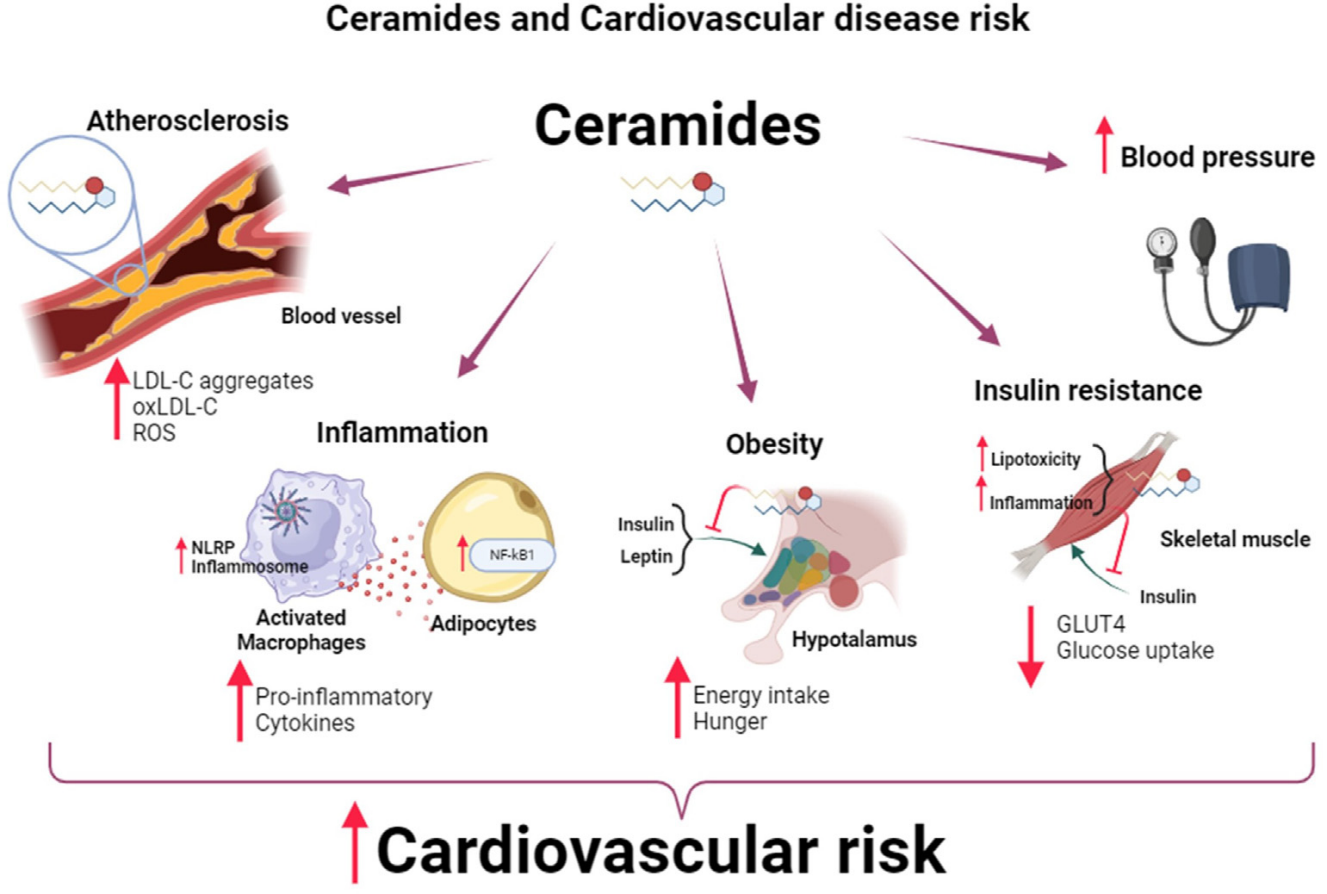
Circulating ceramides and their implications in cardiovascular disease risk. Ceramides, particularly C16:0 and C18:0 long-chain ceramides, play a pivotal role in the pathogenesis of cardiovascular diseases (CVDs). Among the mechanisms underpinning their impact on CVD risk, ceramides can promote atherosclerosis by accumulating within the atherosclerotic plaque where they induce LDL-cholesterol aggregation and oxidation, thereby contributing to oxidative stress. Ceramides induce macrophage polarization toward an M1 proinflammatory phenotype via the activation of the NLRP3-inflammosome, whereas in the adipocytes they trigger the activation of the NF-kβ signaling pathway. Moreover, long-chain ceramides are implicated in the pathogenesis of obesity and type 2 diabetes, by promoting hypothalamic dysfunction and insulin resistance, respectively. Finally, high levels of circulating ceramides may increase blood pressure. GLUT4, glucose transporter-4; NLRP3, nucleotide-binding oligomerization domain-, leucine-rich repeat-, and pyrin domain-containing protein 3; oxLDL-cholesterol, oxidated low-density lipoprotein cholesterol; This figure was created using BioRender.com.

In terms of the mechanisms underpinning the involvement of ceramides in the pathogenesis of atherosclerosis, oxidative stress and inflammation may be pivotal. Indeed, although on one hand, these pathophysiological processes intervene in atherosclerosis onset and progression, they are also triggered by ceramides [17]. In line with this, ceramides influence the activity of the nucleotide-binding oligomerization domain, leucine-rich repeat-containing protein 3 (NLRP3) inflammasome in macrophages and adipocytes, increasing the synthesis of proinflammatory cytokines [18]. In addition, ceramides are involved in triggering endothelial dysfunction by promoting the formation of reactive oxygen species (ROS) and interfering with the production of nitric oxide by the endothelial nitric oxide synthase [13], thereby determining an increase in oxidative stress and hampering vasodilation (Figure 1).

Ceramides seem to play a decisive role in the development of other cardiovascular disease risk factors besides atherosclerosis, such as systemic hypertension, obesity, and T2D. For example, patients with hypertension have higher levels of circulating ceramides that are reduced when normalizing the pressure values with antihypertensive drugs [10]. In keeping with this, a study conducted by Spijkers et al. [19] showed that the baseline concentration of ceramides was higher in isolated carotid arteries of spontaneously hypertensive rats, compared with their normotensive counterparts. Sortilin, a transmembrane receptor deputed to intracellular trafficking, appears to play a role in the etiology of hypertension by dysregulating sphingolipid/ceramide homeostasis and leading to ROS overproduction, thereby altering endothelial-dependent dilation [20]. High blood pressure determines over time a remodeling in heart structure and functionality, ultimately leading to heart failure (HF). However, the inhibition of SPT and the consequent decrease in C16:0, C24:1, and C24:0 ceramide levels in cardiomyocytes prevented the deleterious consequence of hypertension in the heart [21] (Figure 1).

Evidence from animal models also supports the role of ceramides in the pathogenesis of obesity, with this effect being dependent upon the disruption of hypothalamic control of energy balance (Figure 1). Indeed, the intracerebroventricular administration of a permeable ceramide not only resulted in C16:0 ceramide accumulation within the ventromedial hypothalamus but also led to hypothalamic inflammation, a decrease in energy expenditure, and body weight gain in rodents [22]. The involvement of ceramide metabolism in the pathogenesis of obesity is further corroborated by studies demonstrating that an increase in hypothalamic ceramide levels mediated the orexigenic effects of ghrelin, whereas a decrease was crucial for the anorexigenic effects of leptin within the hypothalamus [23]. Diet is also able to shape hypothalamic ceramide accumulation, suggesting these sphingolipids are a potential mediator of the detrimental effects of a high-fat diet (HFD)-induced obesity [24]. In keeping with this, the obesogenic effects of an HFD have been shown to be associated with the accumulation of ceramides within the hypothalamus, further supporting the role of these sphingolipids in hampering the regulation of energy homeostasis [25]. Ceramide accumulation within the hypothalamus also negatively impacts glucose metabolism. In line with this, the inhibition of hypothalamic ceramide synthesis was sufficient to restore insulin sensitivity within the hypothalamus and improve peripheral glucose homeostasis [26]. In agreement with this, it is not surprising that ceramides are also involved in the pathogenesis of T2D. Indeed, lipotoxicity, referred to as the ectopic accumulation of lipids in tissues not suited for fat storage, such as the skeletal muscle and the liver, has been implicated in insulin resistance [27]. However, not all lipids have been shown to be metabolically detrimental, with ceramides and diacylglycerols carrying the blame for the deleterious effects of lipotoxicity (Figure 1). They appear to be “lipotoxic” and contribute to insulin resistance at the muscle level [28,29]. According to Summers et al. [30], ceramides themselves can reduce insulin-induced translocation of glucose transporter protein type-4 onto the plasma membrane of adipocytes and impair skeletal myotubes protein kinase B (AKT) phosphorylation [31], thereby decreasing insulin-stimulated glucose uptake. These effects are mediated by the ability of intracellular ceramides to hamper AKT phosphorylation and activation which, in turn, is a key node in the insulin signal transduction pathways [32]. Moreover, ceramide buildup also promotes insulin resistance in skeletal muscle by impairing insulin receptor substrate-1 via 2 distinct pathways, one involving the protein-kinase-R/c-Jun N-terminal kinase (PKR/JNK) signal [33] and the other relying on Pbx-reculation-protein-1/p160 [34]. In further support of the role of ceramides in insulin resistance, the inhibition of their de novo synthesis using myriocin in genetically obese (ob/ob) and diet-induced obese rats, led to an overall improvement in insulin sensitivity [35]. Nevertheless, not all ceramides have been reported to impair insulin signaling. Indeed, in murine models, C18:0 ceramide appears to be the main driver of insulin resistance [36]. However, this remains controversial, with other reports pointing to C16:0 ceramide as a key driver of insulin resistance [37]. This remains a matter of debate also in humans. Indeed, although higher skeletal muscle content of C18:0, but not total ceramide level, has been observed in obese and T2D volunteers compared with healthy athletes, and associated with insulin resistance [38], in the adipose tissue the de novo synthesis of C16:0 ceramide was a major driver of insulin resistance [39]. Nevertheless, independently of the ceramide subspecies responsible for the cardio-metabolically deleterious effects of these sphingolipids, deranged ceramide homeostasis appears to be a key player in CVD by contributing to the pathogenesis of atherosclerosis, hypertension, obesity, and insulin resistance (Figure 1).

### Ceramides as emerging biomarkers for cardiovascular disease

Ceramides can be reproducibly and consistently assayed and detected in different body fluids, including blood, synovial fluid, and cerebrospinal fluid, which makes them a suitable disease biomarker. In agreement with this, ceramides have been investigated as possible clinical biomarkers for a variety of pathological conditions such as cancer, T2D, Alzheimer’s disease, coronary artery disease (CAD), multiple sclerosis, and depressive disorder [40]. Plasma ceramide levels may predict CVD even more accurately than traditional biomarkers at least in patients with known atherosclerotic CVD [41]. For example, it has been shown that an increase in the circulating concentrations of specific ceramides (C16:0, C18:0, and C24:1) and a low concentration of C24:0 ceramide was associated with increased cardiovascular mortality [41] (Table 1 [[37], [38], [39],[42], [43], [44], [45], [46], [47], [48], [49], [50]]). Furthermore, besides the circulating levels of the aforementioned specific ceramides, their ratio has also been proposed to stratify CVD risk. Indeed, an increase in C16:0/C24:0, C18:0/C24:0, and C24:1/C24:0 ceramide ratios, has also been associated with the incidence of cardiovascular events [41,51] (Table 1 [[37], [38], [39],[42], [43], [44], [45], [46], [47], [48], [49], [50]]).

**TABLE 1:** Ceramide subspecies and ratios as potential biomarkers in the clinical practice

Ceramides subtype		Clinical implications
C14:0		Hepatic steatosis in adolescents [50]
C16:0, C18:0, C24:1		Endothelial dysfunction (C16:0) [43], heart failure [47,48], MACE [44, 45], CAD in T2D [49], plaque vulnerability [44,48], and death [38,44]
C20:0, C20:1, C22:1		Heart failure [47,48]
C24:0		CVD risk factors as age and smoking [42], heart failure [47], MACE [45], plaque vulnerability [48], and CVD death [38]
C24:0		Endothelial dysfunction [43]
Ceramides ratios		Clinical implications
C16:0/C24:0, C18:0/C24:0		CVD mortality in CAD patients [37] and CVD incidence [38,39]
C24:0/C16:0		CVD risk factors as age/smoking [42], heart failure progression and death [47]
Sphingosine-1-phosphate/C24:1, C24:0/C24:1		Ischemic stroke and TIA [46]
C24:1/C24:0		Endothelial dysfunction [43], CVD mortality in CAD patients [37], and CVD incidence [38,39]

Abbreviations: CAD, coronary artery disease; CVD, cardiovascular disease; MACE, major adverse cardiovascular events; TIA, transient ischemic attack; T2D, type 2 diabetes mellitus.

Specifically, ceramides and their ratios are altered in the presence of CVD risk factors, such as T2D and endothelial dysfunction, but also during the progression of the atherosclerotic disease (Figure 1). Ceramides hide the potential to predict T2D [42,43)] and higher levels of C16:0, C18:0, and C24:1 are detected in patients with CAD and T2D, compared with higher C24:0 in CAD patients without significant comorbidities [44]. Peterson et al. [45] demonstrated that the plasma concentration of ceramide C24:0 and the ratio between C24:0/C16:0 ceramides were inversely associated with risk factors for CVD (for example, age and smoking habits) and with the incidence of CAD and HF.

Finally, endothelial dysfunction, which is crucial in the pathogenesis of atherosclerotic disease, has been associated with an increase in ceramides C16:0, C24:0, and C24:1/24:0 ratios suggesting a role for these ceramide subspecies and their ratios in the earlier stages of atherosclerosis development [52].

Ceramides offer a good predictive capability for cardiovascular mortality, allowing identification of patients requiring further therapeutic interventions [53]. Elevated ceramide ratios (C16:0/C24:0, C18:0/C24:0, and C24:1/C24:0) display an association with CVD mortality in CAD [40]. Indeed, higher levels of C16:0, C20:0, C20:1, and C24:0 ceramides in cardiovascular tissue have been observed in both acute and chronic CAD and are positively correlated with the severity of coronary stenosis [10,46]. This also held true for major adverse cardiovascular events (MACE) which were independently associated with the plasma levels of C16:0, C18:0, and C24:1 ceramides in patients with and without CAD [54], whereas C24:0 displayed an inverse relationship with MACE [53]. Furthermore, the same ceramides were associated with vulnerable coronary plaque in subjects with acute coronary syndrome [47] and in patients with ST-segment—elevation myocardial infarction, C16:0, C18:0, and C24:10—ceramides were all predictors of thin-cap fibroatheroma [48]. This novel ceramide score was then validated in the FINRISK 2002 cohort, determining a model with a better predictive capability when including ceramides along with traditional risk factors, such as C-reactive protein, even in patients without previous history of CVD [55]. Ischemic stroke, included among MACE, may also benefit from ceramide assessment for its prediction. Indeed, a rise in specific ceramides might help in recognizing ischemic stroke even when not clearly evident at imaging, in particular when dealing with transient ischemic attacks. In particular, the Sphingosine-1-phosphate/C24:1 and C24:0/C24:1 ratios were proven as sensitive biomarkers in recognizing ischemic stroke, with the first being high even in patients suffering from transient ischemic attacks [56]. Furthermore, long-chain ceramides, such as C16:0 and C18:0 ceramides, rise in plasma following an ischemic stroke and the levels of C18:0, C20:0, and C22:0 ceramides correlate with worse outcomes [49].

Ultimately, ceramides might assume a role even in detecting potential complications of CAD . Elevated levels of ceramides C16:0, C20:1, and C24:1 were observed in the myocardium and serum of patients with HF, despite total ceramide levels remaining unaltered or even decreasing once left ventricular function improved [57]. An opposite behavior was described for C24:0 ceramide, suggesting the possibility to use the ratio C24:0/C16:0 for predicting left ventricular function, HF risk, and mortality [57]. From subsequent analysis by Wittenbecher et al. [58] of the PREDIMED trial, ceramide C16:0 was found to be associated with the risk of developing HF.

For all these reasons, scores implemented with ceramides are actually predictive for CVD endpoints [59]. This is why the Mayo Clinic recently introduced a diagnostic test that quantifies plasma ceramides to identify patients at risk of MACE [2]. These findings lead to the idea of implementing risk detection by taking into account these peculiar lipid molecules for prognosis. One example is the Coronary Event Risk Test (CERT1 including 3 ceramides and 2 ceramide/ceramide ratios subsequently optimized into CERT2 based on ceramides and phosphatidylcholine), able to reliably stratify MACE in patients with stable CAD [60]. However, despite the multiple evidence of the positive association between ceramides and CVD, the role of specific subspecies is still equivocal in this context. For instance, the role of C24:0 ceramide remains controversial being associated both positively with endothelial dysfunction, myocardial infarction with a time-dependent pattern [46] and negatively with cardiovascular mortality [52,61] (Table 1 [[37], [38], [39],[42], [43], [44], [45], [46], [47], [48], [49], [50]]).

## The Effect of Nutrients on Circulating and Tissue Levels of Ceramides

As already anticipated, diet plays a crucial role in shaping cardiovascular health; therefore it is not surprising that it may also modulate ceramide homeostasis. With this regard, although some nutrients and dietary patterns may promote ceramide synthesis, others may counter the accumulation of these sphingolipids, both in the circulation as well as in tissues. Considering fatty acids being a key component of ceramide structure, they represent pivotal dietary components able to modulate ceramide synthesis. The role of dietary lipids in fostering ceramide synthesis has been confirmed in several animal studies focusing on the effect of HFDs on ceramide homeostasis, where HFDs are typically lard-based with ∼50 percent of lipid-deriving energy being in the form of SFA. In keeping with this, it has been shown that HFDs were able to increase circulating, as well as tissue ceramide levels, in animal models. Indeed, an increase in ceramides C16:0 and C22:0 [62,63] was reported in the liver, whereas an induction of ceramide C18:0 was detected in the skeletal muscle of high-fat-fed rodents [[64], [65], [66]].

**FIGURE 2:**
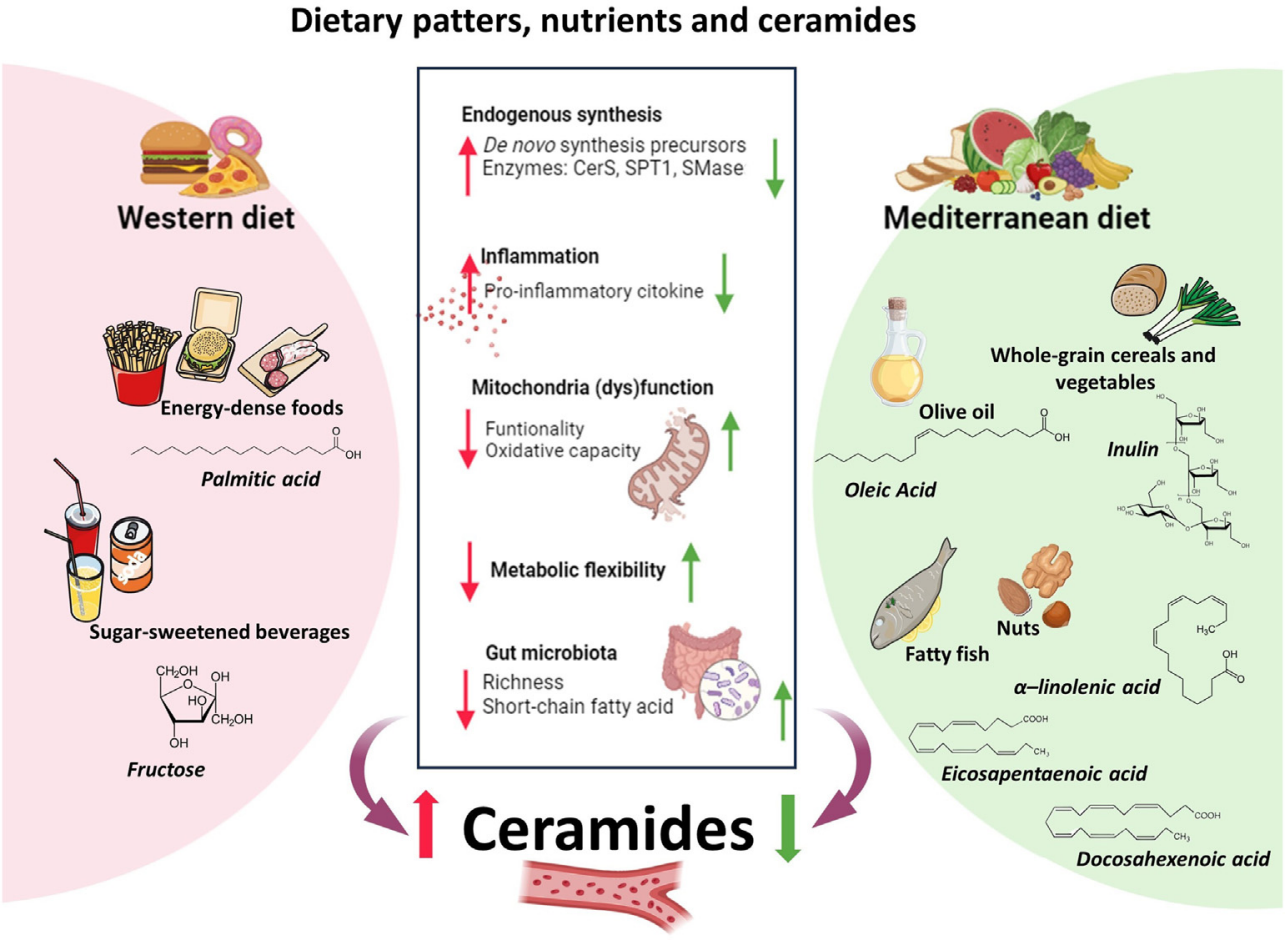
The impact of dietary patterns and nutrients upon circulating ceramide metabolism. Dietary patterns, as well as specific nutrients, can modulate ceramide synthesis and accumulation. The Western diet is characterized by the overconsumption of energy-dense foods rich in long-chain saturated fatty acids (LCSFAs), among which palmitic acid represents the building block for de novo synthesis of these sphingolipids. Moreover, LCSFA promotes proinflammatory responses which can foster the activation of the key enzymes involved in the synthesis of ceramides, both via the de novo and sphingomyelinase pathways. In keeping with this, LCSFA may impair mitochondrial oxidative capacity and functionality with the consequent accumulation of fatty acids which be channeled toward ceramide synthesis. Apart from palmitic acid, also fructose may promote ceramide synthesis. Contrarily to the Western diet, the Mediterranean diet, especially for its content in mono- (MUFA) and omega-3 PUFA, may reduce circulating ceramides by improving mitochondrial oxidative capacity, and metabolic flexibility and reducing systemic inflammation. Furthermore, ω-3 fatty acids, along with inulin, may improve the gut microbiota functionality and diversity as indicated and increase its richness and short-chain fatty acid production. CerS, ceramide synthase; SMase, sphingomyelinase; SPT1, serine-palmitoyl transferase-1. This figure was created using smart.servier.com and BioRender.com.

The most reasonable mechanism linking lard-based HFDs and ceramide accumulation is through the increased supply of ceramide building blocks, particularly in the form of long-chain saturated fatty acids (LCSFAs) [67]. In fact, HFD induces de novo synthesis of ceramides due to the constant supply of palmitic acid, which, alongside serine, represents the building block for the de novo synthesis of ceramides (Figure 2). Apart from providing the substrates that make up the ceramide backbone, HFD also upregulates key enzymes involved in ceramide biosynthesis such as SPT1 [68] and CerS [69] (Figure 2). With this regard, the SFA overload due to HFD consumption promoted endoplasmic reticulum (ER) stress in animal models with liver steatosis, with a concomitant CerS6 upregulation [70] which exacerbated the ER stress, possibly via its implication in C16:0 ceramide synthesis. Ceramide C16:0 belong to long-chain ceramide which was associated with increased cardiovascular mortality [37], whereas very long-chain ceramides, such as C22:0 and C24:0, and particularly the C24:0/C16:0 ratio inversely correlated with CAD risk [45]. Another potential mechanism underpinning the effects of HFD on ceramide accumulation is via the increase in sphingomyelin production in the liver (Figure 2). In this regard, diet-induced de novo synthesis of sphingomyelins increases the secretion of VLDL and LDL enriched with sphingomyelins, which represent a substrate of LDL-SMase. Thus, an enrichment in sphingomyelins within LDL and its consequent catabolism by SMase foster ceramide formation within these lipoproteins, leading to an increase in their aggregability and oxidation [71]. Other potential mechanisms linking high-fat feeding and ceramide buildup within tissues and circulation rely on the modulation of mitochondrial oxidative capacity. In line with this, nutrient overload, and particularly an excessive consumption of LCSFA, seems to be pivotal in eliciting mitochondrial dysfunction which results in decreased oxidative capacity and functionality [72] (Figure 2). As a consequence, catabolic processes such as β-oxidation become impaired [73]. This, in turn, translates into an increase in the bioavailability of fatty acids, such as palmitic acid, which instead of being funneled toward β-oxidation are channeled toward anabolic pathways, including ceramide synthesis [27].

However, the ability of an HFD to promote ceramide synthesis is not only dependent on lipid overload but also upon the quality of the fatty acid consumed. In support of this notion, an HFD enriched in SFA, as opposed to unsaturated fatty acids, significantly increased the ceramide content in cardiomyocytes and skeletal muscle in mice, [74] and in the liver in rats [75]. Noticeably, cholesterol-enriched diet was also able to increase circulating ceramide levels in rats and for some ceramide subspecies, namely C24:0, C24:1, and C24:2, even more than diets enriched with fats from lard. Nevertheless, circulating as well as adipose tissue total ceramides remained higher in the HFD-fed group, with no effect being observed on liver ceramides [76]. The length of the fatty acid that makes up the triglycerides (TG) introduced with the diet differently impacts both de novo ceramides synthesis and sphingomyelin hydrolysis pathways. In support of this, an HFD enriched with medium-chain TG, compared with a standard lard-based HFD, downregulated the expression of both CerS6 and sphingomyelin phosphodiesterase-3 which are implicated in ceramide synthesis, with a consequent reduction in liver ceramide content and enhanced insulin sensitivity. Moreover, the supplementation of an obesogenic diet with LCSFA as opposed to medium-chain saturated fatty acids, while maintaining the constant supply of poly- (PUFA) and MUFA, increased liver steatosis, and plasma proinflammatory ceramides [77]. In terms of the mechanisms underpinning the ability of medium-chain fatty acids to counter ceramide accumulation, these may rely on an increase in oxidative metabolism. Medium-chain fatty acids have been reported to act as signaling molecules able to increase the adenosine monophosphate/adenosine triphosphate ratio, leading to the activation of adenosine monophosphate-activated protein kinase with consequent inhibition of enzymes involved in anabolic pathways, including ceramide synthesis, and activation of β-oxidation [78].

Thus, lipid, and particularly LCSFA, overfeeding in animal models appears to affect circulating ceramide levels as well as its accumulation in metabolically active tissues [79]. However, the relationship between a diet rich in SFA and ceramide levels in real-life patients appears less clearly defined compared with the aforementioned animal models. Indeed, there are some controversial findings linking the overconsumption of SFA and the increase of total plasma ceramides. For instance, a cross-sectional study with 2860 participants showed that SFA intake was directly associated with higher circulating concentrations of 16:1;O_2_ sphingolipids including ceramides and their derivatives (sphingomyelins and sphingosine 1-phosphates) [80], whereas a study on eleven T2D subjects consuming an isocaloric HFD over 3 wk, failed to alter ceramide levels in the skeletal muscle and insulin sensitivity compared with a carbohydrate-rich diet [81]. The inability of the fat-rich diet to increase ceramide levels within the skeletal muscle may be dependent upon the increase in adiponectin levels induced by this dietary regimen and the fact that the diets used as part of this study were isocaloric [81]. Surprisingly, in this study, adiponectin levels resulted to be increased after 3-wk of lipid overfeeding. Because adiponectin represents an adipokine that can stimulate fatty acids oxidation and mitochondrial biogenesis, its increase may represent a compensatory mechanism triggered by increased fat intake [81]. However, when considering human studies, there are factors other than diet, which may influence ceramide homeostasis. Of these, intramyocellular lipid droplet size and location may impact the ability of lipid overfeeding to induce ceramide accumulation in human skeletal muscle. Indeed, it has been shown that the amount of small peripheral intramyocellular lipid droplets was associated with increased lipid oxidation efficiency in response to overfeeding. This, in turn, resulted in a reduced accumulation of lipotoxic species precursors responsible for ceramide synthesis, as well as reduced insulin resistance [82].

As already described, LCSFAs appear to be the primary drivers underpinning ceramide synthesis, particularly in animal models and when provided as part of hypercaloric diets. On the contrary, other types of fatty acids, such as MUFA and PUFA, have not been reported to promote ceramide synthesis. Moreover, in some cases, they seem to counter LCSFA-induced ceramide accumulation.

In particular, dietary fish oil reduced specific plasma ceramide subspecies (C16:0, C22:0, C24:0, and C26:0) and lowered blood pressure and circulating thromboxane, thereby improving endothelial function [83] (Figure 2). However, fish oil supplementation increased C20:0 and C24:1 glucosylceramides [83]. Similar effects were appreciated in tissues, where omega-3 PUFAs attenuated the rise in C18:0 ceramide-induced by an LCSFA-enriched HFD in the skeletal muscle of mice, despite no changes in body weight or triacylglycerols levels being detected between groups [84,85]. The same holds true for the liver, where ω-3 PUFA from fish oil countered the accumulation of ceramides induced by a high-fat and high-sucrose diet [86].

However, the effect of ω-3 PUFA upon ceramide circulating levels and accumulation within tissues seems to be influenced by their quality and sources. For example, in mice, the consumption of krill oil was more effective than fish oil in lowering the hepatic content of ceramides [87]. This effect is in agreement with the hypothesis that krill oil has a more powerful anti-inflammatory effect compared with fish oil [87]. Hence, the consumption of krill oil may be able to reduce proinflammatory cytokines, thus reducing the activation of SMase [87] (Figure 2).

Among PUFA, DHA and EPA are of particular interest for their ability to regulate ceramide levels and synthesis. In mice, the supplementation of DHA and EPA affected total ceramide concentration and quality, specifically by reducing saturated ceramide species and increasing unsaturated ceramide species such as C24:1 in adipose tissue and skeletal muscle, contributing to an improvement in insulin sensitivity and lowering inflammatory cytokines [88] (Figure 2).

Even in human studies, ω-3 PUFA from fatty fish was able to decrease the circulating levels of ceramides, as observed in a pilot study on CAD patients, while lean fish consumption did not significantly affected total circulating ceramides [89]. Besides ω-3 PUFA, other unsaturated fatty acids, such as oleic acid, were reported to counter the effect of saturated fatty acids on ceramide synthesis. In line with this, a lipidomic analysis in women comparing a diet enriched in palmitic compared with oleic acid demonstrated that whereas the first promoted a greater android adiposity and insulin resistance, the second led to a reduction in circulating, as well as skeletal muscle, ceramide levels [90]. Given the anti-inflammatory potential of oleic acid, it is possible that the reduction in ceramide levels may be partially attributable to its ability to lower circulating proinflammatory cytokines, which are known to promote the activation of the enzymes involved in ceramide synthesis [90] (Figure 2).

Despite lipids appearing to represent the primary dietary factor influencing ceramide synthesis, sugar, and fructose in particular, represent an additional nutritional culprit in fostering ceramide synthesis (Figure 2). In agreement with this, the administration of liquid fructose in rodents induced an increase in liver ceramide levels, paralleled by hyperleptinemia and liver leptin resistance possibly caused by ceramide-mediated activation of phosphatase 2A [91]. In addition, the ability of fructose to enhance ceramide accumulation within the liver was reported to occur via its detrimental impact on the gut microbiota [92]. Indeed, the effect of fructose on liver ceramide content was blunted when fructose was administered alongside antibiotics (ampicillin and neomycin) [92].

The gut microbiota may represent a further player linking diet, lipid metabolism, and metabolic health. In keeping with this, a clinical trial on healthy volunteers showed that the consumption of inulin and ω-3 decreased CVD-related circulating ceramide C16:0/C24:0 ratio with this effect being linked to the prebiotic activity of both inulin and ω-3 fatty acids [93] (Figure 2).

Also, an intervention study showed that a low-calorie diet enriched with soluble fibers, high protein intake, and low glycemic index carbohydrates, can increase gene richness in the gut microbiota which was also inversely related to circulating ceramide levels, especially C18:1 [94].

### Dietary patterns and ceramide homeostasis

Besides the effects of single nutrients, ceramides can also be modulated by dietary patterns. Indeed, although the Western diet contains nutrients able to foster ceramide synthesis, namely LCSFA and fructose, the opposite is true for the Mediterranean diet (MD) (Figure 2). The MD is a dietary pattern characterized by low intake of ultra-processed food, LCSFA, and refined sugar which are replaced by high amounts of dietary fiber, unsaturated FA, in particular ω-3 PUFAs and MUFAs, as well as polyphenols which have been shown to improve cardiometabolic health [95]. These nutrients typical of MD have been shown to enhance oxidative metabolism and metabolic flexibility [96] with a consequent improvement in lipid metabolism, which, in turn, is pivotal in shaping cardiovascular health [97]. Hence, it is plausible to believe that the beneficial effects of the MD may also rely on the modulation of ceramide circulating levels. However, the PREDIMED prospective study showed that the MD supplemented with extra-virgin olive oil or nuts decreased CVD risk also in individuals with a high ceramide score, suggesting that the beneficial effects of the MD on cardiovascular health may occur independently of a decrease in circulating ceramides [14]. The MD is a dietary pattern that emphasizes the consumption of fruits and vegetables, which, also in line with their fiber and bioactive content, may play a prominent role in mediating the beneficial effects of the MD on cardiovascular health also by modulating lipid metabolism and ceramide homeostasis. In this regard, the consumption of fruit and vegetables for 8 wk, in agreement with the USDA MyPlate Guideline, promoted a decrease in the levels of circulating C24:0 ceramide which, along with an increase in C16:0 ceramide as well as a decrease in waist circumference, blood pressure, and circulating cholesterol. Conversely to what was expected, these findings suggest a positive association between C16:0 ceramide and an improved metabolic profile. This may suggest the impact of specific ceramide subspecies on metabolic health remains still to be clarified [98].

Finally, besides their effect on lipid metabolism, nutrients or nutraceuticals consumed as part of this dietary pattern may also impact de novo ceramide synthesis indirectly, for example, by downregulating proinflammatory cytokines [98,99] (Figure 2).

The ketogenic diet represents an additional nutritional tool with the potential to modulate ceramide homeostasis and the accumulation of specific subspecies. In mice, a ketogenic diet, indeed, downregulated CerS6 while upregulating CerS2, an effect that prevented the accumulation of harmful long-chain ceramides (C16:0 and C18:0) while increasing the accumulation of protective very-long-chain ceramides in the liver [100]. Besides its ability to modulate the expression of key enzymes involved in ceramide synthesis, the ketogenic diet may impact ceramide homeostasis by its known capacity to boost fatty acid catabolism [101], mitochondrial biogenesis [102] and reduced inflammation [103].

Similarly to the ketogenic diet, caloric restriction was shown to decrease ceramide content in the liver, with this effect being enhanced by dietary supplementation of proteins and calcium [104]. However, caloric restriction effects on ceramide levels seem to be tissue-specific. For example, caloric restriction did not affect overall myocardial ceramide levels in mice but rather lowered C20:0 and C22:0 ceramides with a consequent attenuation in myocardial lipotoxicity [105].

Thus, although diet may play a prominent role in shaping ceramide homeostasis, evidence on the ability of dietary patterns to modulate circulating ceramide levels and type remains spare. To the same extent, whether the effect of diet on cardiovascular health occurs via the modulation of ceramide metabolism remains to be elucidated.

Finally, ceramides can also be consumed through supplements intended for skin and hair care [106,107]. However, to date, there is no evidence of the role of ceramide-containing supplements on cardiovascular health. In addition, it should be considered that the putative impact of these supplements on CVD depends on the type and levels of the ceramides they contain.

## Conclusions

Ceramides are emerging as key actors in the pathogenesis of CVD, also considering their ability to elicit inflammatory responses and contribute to the development of obesity and insulin resistance. In addition, their role as novel biomarkers able to independently predict cardiovascular events, surge ceramides as a powerful and innovative diagnostic tool to identify individuals at high cardiovascular disease risk. However, the predictive power of these sphingolipids toward cardiovascular events appears to be specific to C16:0, C18:0, and C24:1 ceramides which have been reported to describe the risk of cardiovascular events independently of other well-defined risk factors [2,12]. Ceramides are also at the nexus between diet and CVD risk. Indeed, the same nutrients and dietary patterns that have been widely shown to negatively impactCVD risk, namely LCSFA, fructose, and the Western diet, are also able to promote ceramide synthesis, as suggested by animal and human studies. On the contrary, nutrients able to counter ceramide synthesis, such as ω-3 PUFAs, have also been associated with a decrease in CVD risk. Nevertheless, the effects of these nutrients on the inhibition of ceramide synthesis are mainly limited to animal studies. However, despite ceramide metabolism appearing sensitive to diet and these sphingolipids being increasingly implicated in CVD risk, it remains to elucidate whether ceramide may represent a nutritional target to improve cardiovascular health. In light of this, further studies are warranted to elucidate whether nutritional interventions can directly modulate the circulating and tissue ceramide profiles and if this translates into an improvement in cardiometabolic health. Particularly, based on the available data in cell and animal models, these studies should rely on nutritional approaches that on one hand decrease the supply of the building blocks for ceramide synthesis (that is, LCSFA) while promoting a shift in lipid metabolism toward catabolic pathways, specifically β-oxidation. These studies may shed light on the role of circulating ceramides as prognostic markers to monitor the effectiveness of nutritional interventions on cardiovascular health. To conclude, despite these research gaps remaining to be completely elucidated, ceramides still represent a promising biomarker at the interphase between diet quality and cardiovascular health as well as a putative nutritional target to lower CVD risk.

## Author contributions

The authors’ contributions were as follows – DS, AP: conceptualized the review; RS, SA, ADV, GS, SM, VF, SF, DS: wrote the first draft of the review; FC, JMS, AP: critically revised the manuscript and contributed to writing the final draft of the review; SA draw the figures; and all authors read and approved the final manuscript.

## Conflict of interest

The authors declare no conflict of interest.
